# Genome-Wide Dissection of Sorghum B3 Transcription Factor Family Identifies SbLAV1 as a Critical Transcriptional Regulator of Starch Biosynthesis in Developing Sorghum Grains

**DOI:** 10.3390/plants14111701

**Published:** 2025-06-03

**Authors:** Xiangling Gong, Jing Li, Zheyu Yan, Anqi Sun, Yi Zheng, Min Yin, Qianlin Xiao, Zhizhai Liu

**Affiliations:** College of Agronomy and Biotechnology, Southwest University, Chongqing 400715, China; 15098024284@163.com (X.G.); lijing1923@126.com (J.L.); 13786041673@163.com (Z.Y.); 18090310071@163.com (A.S.); 17823263414@163.com (Y.Z.); 18584519883@163.com (M.Y.)

**Keywords:** Sorghum (*Sorghum bicolor* L.), B3 transcription family, *SbLAV1*, transcriptional regulation, starch biosynthesis

## Abstract

Sorghum (*Sorghum bicolor* L.) is the fifth largest cereal crop in the world and widely used in the fields of food, feed, brewing, and fuel, while knowledge is mostly limited for sorghum grain development, including starch biosynthesis. B3 family transcription factors (TFs) play a crucial role in plant development, including grain development, dormancy, and storage of nutrients. In the present study, a comprehensive analysis of sorghum B3 genes was performed, and a total of 76 related genes were identified to be distributed on 10 chromosomes across the whole sorghum genome. According to the sequence features, the sorghum B3 family members were divided into four sub-families of ARF, RAV, LAV, and REM. Multiple elements, i.e., light-responsive elements, phytohormone-responsive elements, growth and development-related elements, and stress-responsive elements, were discovered to be located within the 2000 bp upstream of the translation start site. Results of expression analysis across multiple tissues suggested significantly different expression patterns of sorghum B3 genes. Further assays confirmed that *SbLAV1*, which belonged to the LAV subfamily of B3, co-expressed with 15 key starch biosynthesis-related genes (SBRGs), and the corresponding product of SbLAV1 could activate the promoter activities of multiple key SBRGs. Collectively, the integrative results of the present study indicate that B3 family members, including SbLAV1, might play critical roles in starch biosynthesis and grain development in sorghum.

## 1. Introduction

Sorghum (*Sorghum bicolor* L.) originated in Africa over 5000 years ago, and it is recorded in FAO as a vital cereal crop worldwide, with production exceeding 60 million metric tons. Sorghum grains contain abundant starch reserves, typically constituting approximately 70% of grain weight, with multifaceted applications in the fields of food, feed, brewing, and fuel [1]. Sorghum boasts greater resilience and adaptability than other cereal crops, thriving across a wider geographical range [2]. As a dietary staple for over 500 million people in arid and semi-arid regions across the world, sorghum plays a vital role in safeguarding regional food security [3,4]. Meanwhile, Sorghum also exhibits itself as a pivotal reference organism in functional genetics and genomics research, particularly for tropical C4 grasses, owing to its compact diploid genome, high phenotypic diversity, and close evolutionary relationship with other economically vital cereals [1,3,5].

Starch, the most important storage carbohydrate in plants, is widely used in human food, feed, and industrial raw materials [6,7]. Despite scientific breakthroughs in cell-free chemoenzymatic catalytic synthesis of starch from CO_2_ [8], starch obtained for mankind remains predominantly dependent on plant photosynthesis. Starch biosynthesis occurs in the plastid of higher plants through a coordinated enzymatic cascade comprising ADP-glucose pyrophosphorylase (AGPase), starch synthases (SSs), starch-branching enzymes (SBEs), debranching enzymes (DBEs), and starch phosphorylase (SP) [9,10]. AGPase governs the biosynthesis of ADP-glucose (ADPG), and Brittle1 (BT1) facilitates ADPG transmembrane transport into amyloplasts. These two components collectively provide the direct substrate donor for glucan chain elongation during starch biosynthesis [6,9,11]. The elongation of polyglucan chains is primarily mediated by SSs, which can be categorized into two distinct isoforms: granule-bound starch synthase (GBSS) and soluble starch synthase (SSS). GBSS and SSS coordinately determine the structural characteristics and chain length distributions of glucan polymers [9]. SBEs and DBEs are mainly responsible for the formation of correct branching chains and starch granule structures [12,13]. SP plays a pivotal role in the assembly of functional enzyme complexes and the initiation of starch biosynthesis [14,15]. Although multiple enzymes are coordinated to participate in starch biosynthesis, they are regulated at multiple hierarchical levels, including phosphorylation [16,17], protein-protein interactions [18], allosteric regulation of enzymatic activity [19], and transcriptional regulation [20].

In cereal crops, transcriptional regulation is an important mechanism for starch biosynthesis in grains [20]. For example, OsRSR1 [21], OsbZIP58 [22], OsNAC20/26 [23], and OsSGL [24] function as transcriptional regulators that coordinate the expression of starch biosynthesis-related genes (SBRGs), thereby mediating starch production in rice grains. ZmbZIP91 [25], ZmEREB156 [26], ZmMYB14 [27], ZmDof3 [28], and ZmNAC34/126/128/130 [29,30,31] regulate starch biosynthesis by transcriptionally controlling key SBRGs in maize kernels. TaNAC019-A1 [32], TubZIP28 [33], and TaNAC-A18 [34] play important roles in starch biosynthesis in wheat grains. *HvSUSIBA2*, a pivotal transcriptional regulator of starch biosynthesis in barley [35], was reported to modulate yield performance when overexpressed in rice [36]. For transcription regulation, *cis*-elements usually serve as combining sites between transcription factors (TFs) and target genes. For instance, sugar-responsive elements within the *iso1* promoter function as direct binding sites for HvSUSIBA2 [35]; the ACTCAT motif is recognized by ZmbZIP91 [25]; ZmNAC126 exhibits binding specificity to tandem CACG repeats [29]; the ACGCAA element was identified as the cognate binding site for ZmNAC128/130 and TaNAC-A18 [31,34], while TaNAC019-A1 binds to the ACGCAG motif [32]; and the AAAG (P-box) and ACGT (O2-box) motifs within the *ZmSSIII* promoter serve as binding platforms for the TFs of O2 and PBF, respectively [37]. In sorghum, it was also documented that the CACGCAA motif was the binding element of endosperm-enriched NAC-type TFs in sorghum grain [38], and the P-Box motif (TGTAAAG) was recognized by SbDOF21 [39]. Therefore, the transcriptional regulation may be an important regulatory mechanism for starch biosynthesis in sorghum grains.

The B3 TF family, characterized by the presence of a conserved B3 domain, encompasses four subfamilies of LAV (LEC2 [LEAFY COTYLEDON2]-ABI3 [ABSCISIC ACID INSENSITIVE3]-VAL), ARF (AUXIN RESPONSE FACTOR), RAV (RELATED TO ABI3 and VP1), and REM (REPRODUCTIVE MERISTEM) and represents a crucial group of TFs in plants [40]. LAV subfamily member LEAFY COTYLEDON 1 (LEC1) orchestrates the complete development from early embryogenesis to late seed maturation. LEC2, ABI3, and FUS3 also exhibit specific binding capabilities to *cis*-elements, including the RY motif of CATGCA, G-box motif of GACGTG, and AuxRE motif of TGTCTC, to govern seed development [41]. ARF subfamily members, such as AtARF4 and AtARF3, were mainly reported to participate in plant development through auxin response [42]. *AtRAV1*, a RAV subfamily member, is down-regulated by brassinosteroid and may act as a negative regulator of lateral root and rosette leaf development [43]. REM subfamily members were also reported to regulate flower development [44].

Apart from plant growth and development, B3 TFs also play pivotal roles in regulating the accumulation of storage proteins, lipids, and starch in grains. In *Arabidopsis thaliana*, ABI3 primarily modulates total seed protein content, whereas FUS3 governs lipid biosynthesis [45,46]. *ZmVp1*, the first cloned B3 gene in maize, activates transcriptional regulation of *Galactinol Synthase2* for seed raffinose accumulation [47]. *ZmAFL4*, the ortholog of *AtLEC2*, exhibits preferential expression in pollen and kernels, where it can function to balance carbon metabolism and starch deposition during endosperm development to regulate grain filling [48]. Notably, ZmABI19 coordinates grain filling by modulating transcriptional networks involved in high-abundance endosperm genes and TFs, such as *O2*, *ZmbZIP22*, *NAC130*, and *Opaque11* [49]. However, there are few reports on the biological functions of B3 family TFs in sorghum grains.

In the present study, we conducted genome-wide identification and characterization of the B3 TF family in sorghum to elucidate the potential regulatory mechanisms underlying grain development and nutrient reservoir formation. A total of 76 genes belonging to the B3 family were identified along the sorghum genome. Expression analysis of these B3 genes revealed that LAV subfamily members, i.e., *SbLAV1*, *SbLAV4*, *SbLAV5*, and *SbLAV6*, exhibited seed- and inflorescence-specific genes in sorghum. In addition, co-expression analysis and transient expression assays indicated that AAA proteins were potential active regulators of starch biosynthesis in the developing sorghum grains. The summary results of this study provide valuable information for the B3 family in sorghum and suggest the potential function of B3s in sorghum grains, including starch biosynthesis and other nutrient accumulation.

## 2. Results

### 2.1. Identification of Sorghum B3 Family Members

A total of 76 B3 family members were finally collected from the entire sorghum genome, distributed on all 10 chromosomes (Table S1). The number of amino acids encoded by these 76 sorghum B3 genes ranged from 109 aa (SbREM16) to 1159 aa (SbARF18), with the molecular weight and isoelectric points (pI) ranging from 11.93 kDa (SbREM16) to 128.16 kDa (SbARF18) and 4.75 (SbREM16) to 10.15 (SbRAV13), correspondingly (Table S1). The predicted results of subcellular localization suggested that 58 B3 proteins were located within the nucleus, eight within the chloroplast, and the rest 10 within the cytoplasm (Table S1).

### 2.2. Evolution and Synteny Analysis of Sorghum B3 Family Members

**Figure 1:**
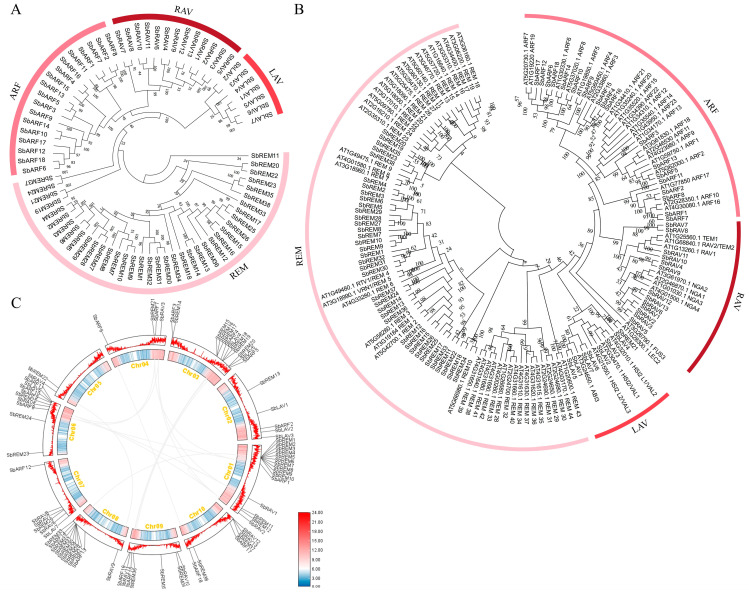
The evolutionary tree and collinear analysis of SbB3s. (**A**) Evolutionary tree of sorghum B3 proteins; bootstrap support values are specified; (**B**) B3 protein tree of *Sorghum bicolor* (Sb) and *Arabidopsis thaliana* (At); bootstrap support values are specified; (**C**) Chromosome localization and collinear map of sorghum B3 genes. Gray lines represent linear gene pairs related to the sorghum B3 gene family. Chr01 to Chr10 refer to all ten chromosomes of the sorghum genome.

76 SbB3s were divided into four subfamilies of ARF (SbARFs), RAV (SbRAVs), LAV (SbLAVs), and REM (SbREMs), with the corresponding subfamily members of 18, 13, 7, and 38 (Figure 1A). The combined B3 proteins tree from two species discovered conserved evolutionary relationships of these proteins between sorghum and *Arabidopsis* (Figure 1B). For example, most REM members of both SbB3s and AtB3s formed separate clades, establishing species-specific REM subclasses. Similar trends were also observed among SbRAVs and AtRAVs (Figure 1B). Differing from the patterns of REMs and RAVs, those of both ARFs and LAVs from sorghum and *Arabidopsis* exhibited non-specific clustering trends (Figure 1B). Specifically, *Arabidopsis* LEC2 and its sorghum ortholog SbREM21 formed an isolated clade, occupying a position outside of any subfamily (Figure 1B). Overall, B3 family genes in sorghum exhibited homologous trends to those of *AtB3*s in *Arabidopsis*.

Chromosome localization of 76 SbB3 genes (*SbB3*s) confirmed that *SbB3*s were mainly distributed in the vicinity of telomeres, where gene abundances were relatively high (Figure 1C). The collinearity analysis of the *SbB3*s revealed 14 collinear gene pairs distributed on eight chromosomes among the members of *SbARF*s, *SbRAV*s, and *SbREM*s (Figure 1C). No collinearity relationships were observed among all seven *SbLAV*s that located on Chr02, 03, 06, and 07 (Figure 1C). These results suggested that, except for the subfamily members of *LAV*, tandem and segmental replication might be the main driving forces for the evolution of the sorghum B3 family.

### 2.3. Conserved Motifs, Structural Domains, and Gene Structure of Sorghum B3s

**Figure 2:**
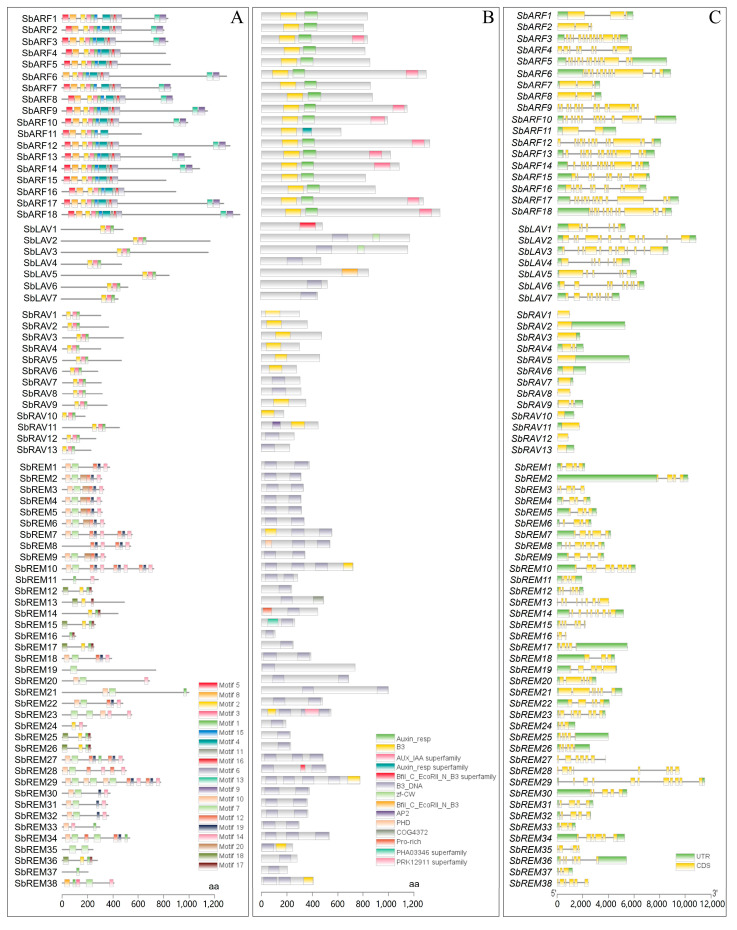
The motif organization, domain, and exon–intron structure of the sorghum B3 family. (**A**) The motif organization of sorghum B3 proteins; (**B**) Domain of sorghum B3 proteins; (**C**) Gene structure of sorghum B3 genes. Different colored boxes in both (**A**) and (**B**) represent the corresponding motifs (**A**) and domains (**B**) of B3 proteins in sorghum, while the green and yellow boxes in (**C**) refer to the UTRs and exons of B3 genes in sorghum, respectively, and the black lines refer to the introns of these genes.

Within subfamilies, analogous motif distribution patterns. Notably, all 18 SbARFs contained 9 out of 20 motifs, i.e., Motif 1/2/3/4/5/8/11/15/16, the same motifs as those observed within 17 SbARFs, except that SbARF11 contained an additional Motif 6 (Figure 2A). All SbLAVs and SbRAVs consistently possessed Motif 1/2/3 that was even arranged in an identical sequential order across all corresponding proteins (Figure 2A). Among 24 SbREMs, Motif 7 and 14 were predominantly observed on the C-terminal, while Motif 10 was detected in 22 SbREMs (Figure 2A).

Furthermore, 14 conserved domains were identified among 76 SbB3s. Notably, B3-type domains, i.e., B3, Bfil_C_EcoRII_N_B3, and B3_DNA, were universally presented across all SbB3s (Figure 2B). SbARFs, SbLAVs, and SbRAVs typically contained only one B3-type domain, whereas SbREMs harbored at least one (Figure 2B). Additionally, subfamily-specific conserved domains were identified, such as SbARFs uniquely possessing the Auxin_resp domain, SbLAVs of SbLAV2 and SbLAV3 containing zf-CW domains, SbRAVs of SbRAV11 featuring an AP2 domain, and SbREMs being characterized as containing Pro-rich domains (Figure 2B). Furthermore, comparative analysis of domain sequences from various subfamilies discovered a high degree of conservation among B3 domain sequences within the same subfamily (Figures S1–S4). All of SbARFs, SbLAVs, and SbRAVs contained conserved fragments within their conserved B3 domains (Figures S1–S3). Similarly, the SbREMs exhibited conserved sites within their conserved B3 domains (Figure S4). In addition, the comparative analysis also showed that B3 domain sequences among different subfamilies exhibited a certain degree of variability.

Gene structure analysis of *SbB3*s revealed substantial divergence among subfamilies while maintaining relative conservation within each subfamily (Figure 2C). The results showed that *SbARF*s exhibited two distribution patterns, where *SbARF1*/*2*/*7*/*8* each contained three exons, while the remaining *SbARF*s possessed more than ten exons each (Figure 2C). *SbRAV*s also possessed two distribution patterns, where *SbRAV4*/*9* contained three exons, and the remaining 11 *SbRAV*s retained only one exon. *SbLAV*s displayed variable exon numbers (6 to 12), and *SbREM*s exhibited remarkable variation in both exon-intron organization (3–11 exons). The diversity of both gene and protein sequences of the sorghum B3 family members also suggested a wide range of their functions. 

### 2.4. Cis-Elements Predicted within the Promoter Regions of SbB3s

**Figure 3:**
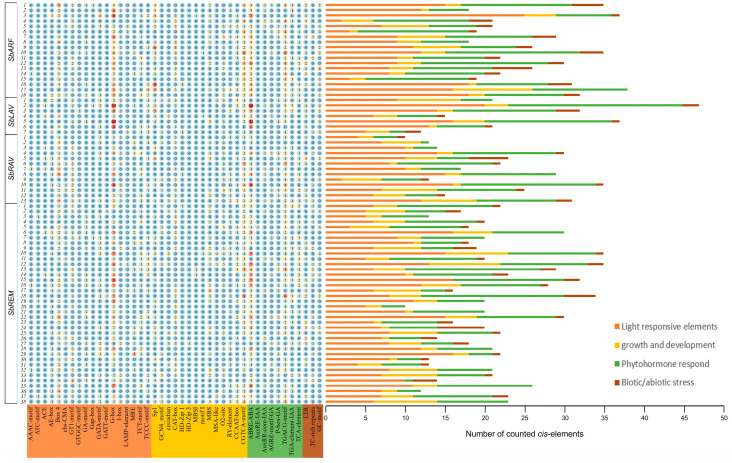
The distribution of *cis*-elements within the promoter regions of sorghum B3 genes.

A total of 43 distinct *cis*-elements were identified within the 2000 bp upstream of TSS, categorized into four functional groups, i.e., light-responsive elements, phytohormone-responsive elements, growth and development-related elements, and stress-responsive elements (Figure 3).

Light-responsive elements constituted the most diverse categories of 19 different types, including G-box, I-box, and AE-box (Figure 3). Phytohormone-responsive elements covered nine major types, predominantly auxin-associated motifs (AuxRE, AuxRR-core, TGA-element), gibberellin-responsive elements (GARE-motif, P-box), salicylic acid-related TCA-element, abscisic acid (ABA)-responsive ABRE, and methyl jasmonate-associated motifs (CGTCA-motif, TGACG-motif) (Figure 3). Growth- and development-related elements comprised 12 types, including CAT-box, HD-Zip1, and MBSI (Figure 3). Meanwhile, stress-responsive elements were primarily represented by low-temperature-responsive *cis*-elements (LTR), defense/stress-inducible *cis*-elements (TC-rich repeats), and anaerobic induction-related *cis*-elements (GC-motif) (Figure 3).

Quantitative analysis revealed distinct enrichment patterns of four groups of *cis*-elements. G-box was the most abundant *cis*-element for light-response, which was detected in the promoter regions of 63 *SbB3*s. Similarly, the ABRE, ABA-responsive element, was also identified within 63 *SbB3*s. Among growth and development elements, CAT-box exhibited the highest frequency, localized to the promoters of 40 *SbB3*s. For stress-responsive elements, LTR was predominant, observed in the upstream regions of 34 *SbB3*s. In summary, according to the various *cis*-elements discovered, *SbB3*s might participate in plant development, light response, hormonal regulation, and various stress responses.

### 2.5. Expression Profiling of SbB3s and Co-Expression Analysis with SBRGs

**Figure 4:**
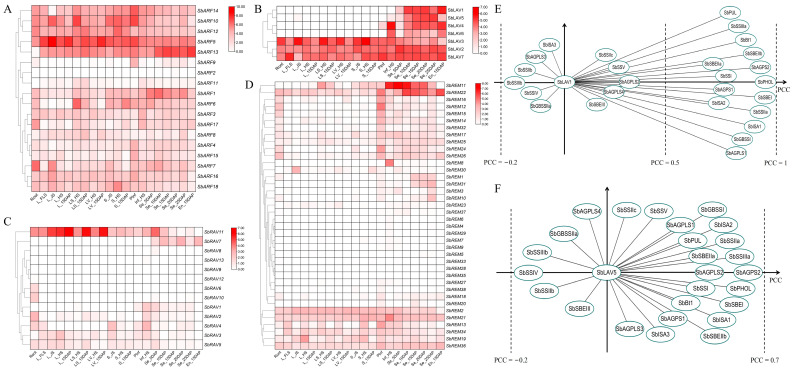
The expression analysis of sorghum B3 genes among different tissues and co-expression analysis with SBRGs. (**A**–**D**) The expression analysis of *SbARF*s (**A**), *SbLAV*s (**B**), *SbRAV*s (**C**), and *SbREM*s (**D**) among different tissues. L, LS, LV, S, Pinf, Inf, Se, and En refer to the corresponding tissues of leaf (L), leaf sheath (LS), leaf vein (LV), stem (S), primary inflorescence (Pinf), inflorescence (Inf), seed (Se), and endosperm (En); while the abbreviations after “_” refer to the developmental stages of 5th developed leaf stage (FLS), jointing stage (JS), heading stage (HS), and 5/10/15/20/15 days after pollination (DAPs); (**E**) to (**F**) Co-expression analysis of *SbLAV1* (**E**) and *SbLAV5* (**F**) with SBRGs.

Based on the RNA-seq of multiple sorghum tissues [38], expression pattern analysis was conducted for 76 *SbB3*s. The results showed that though 18 *SbB3*s exhibited fragments per kilobase of transcript per million mapped reads (FPKM) values less than 1 among all sorghum tissues, *SbB3*s exhibited distinct expression patterns across various tissues within different subfamilies (Table S2, Figure 4). For all *SbARF*s, 17 genes possessed expression signals among all tissues, i.e., *SbARF1* and *SbARF13* had relatively higher expression levels in grains, while *SbARF2* exhibited the contrary trend (Figure 4A). *SbLAV*s were clearly categorized into two types, i.e., four genes of *SbLAV1*/*5*/*4*/*6* were highly expressed in inflorescences and grains, while the other three genes of *SbLAV2*/*3*/*7* were highly expressed in all tissues (Figure 4B). Among *SbRAV*s, *SbRAV11* was highly expressed in leaves, *SbRAV7* exhibited more abundant transcripts in both grains and endosperms, while the rest of the *SbRAV*s possessed moderate to low expression levels within all tissues (Figure 4C). The expression levels of 36 *SbREM*s were classified into three patterns, i.e., two genes of *SbREM11*/*22* that were highly expressed in inflorescences and grains, six genes of *SbREM2*/*21*/*13*/*34*/*19*/*36* that possessed moderate expression levels within almost all tissues, and the rest of the genes with low levels within most or all tissues (Figure 4D).

Based on the expression patterns, *SbLAV1* and *SbLAV5* were identified as grain-specific, highly expressed *SbB3*s in sorghum. Pearson correlation coefficient (PCC) analysis was conducted to examine the co-expression trends between both *SbLAV1*/*5* and SBRGs in sorghum grains. The results showed that *SbLAV1* exhibited PCCs of >0.7 with 15 SBRGs (Figure 4E), while *SbLAV5* possessed PCCs > 0.5 with only four SBRGs of *SbSBEI*, *SbSSIIIa*, *SbPHOL*, and *SbAGPS2* (Figure 4F). These results further demonstrated that *SbLAV1* co-expressed with more SBRGs and potentially functioned to regulate starch biosynthesis in sorghum grains.

### 2.6. RT-qPCR Based Expression Analysis of SbB3s in Developing Sorghum Grains

**Figure 5:**
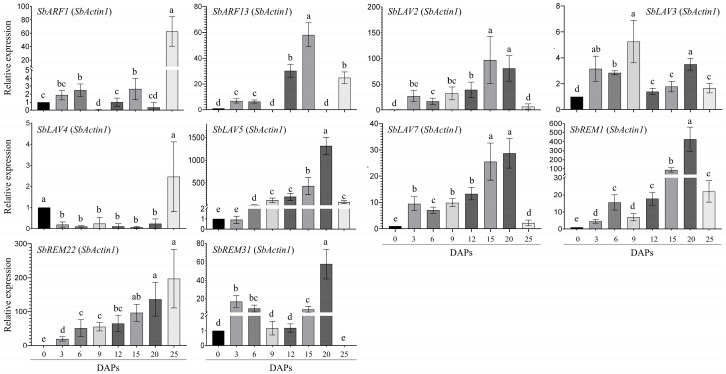
The RT-qPCR analysis of sorghum B3 genes in sorghum grains at different development stages by using *SbActin1* as an internal. Different letters on the bars refer to significance at *p* < 0.05, while the same letter refers to non-significance.

According to the relatively abundant transcripts *via* RNA-seq, 10 *SbB3*s were screened and further validated through RT-qPCR under two internal controls of *SbActin1* and *SbEif4α* among the developing sorghum grain samples from 0 day after pollination (DAP) to 25 DAPs. The results showed that, except for *SbLAV3*, the other nine genes were highly expressed in sorghum grains from middle to late developing stages, i.e., 15 DAPs to 25 DAPs under both controls (Figure 5 and Figure S5). Especially, the expression levels of five genes of *SbLAV2*/*5*/*7* and *SbREM1*/*22* gradually increased from 0 DAP to 20 DAPs, with remarkable high points at 15 and 20 DAPs, and then decreased to very low levels at 25 DAPs, except for *SbREM22*, which exhibited even higher expression levels at 25 DAPs (Figure 5). Similar to that of *SbREM22*, two other genes, *SbARF1* and *AbLAV4*, also exhibited the highest expression levels at 25 DAPs (Figure 5). Relatively higher expression levels were observed for both *SbLAV3* and *SbREM31* at early (3, 6, or 9 DAPs) and late stages (20 or 25 DAPs), while higher expression levels were only observed for *SbARF13* at 12, 15, and 25 DAPs; lower or even no expression signals were observed at other stages (Figure 5).

### 2.7. Functional Properties of SbLAV1

**Figure 6:**
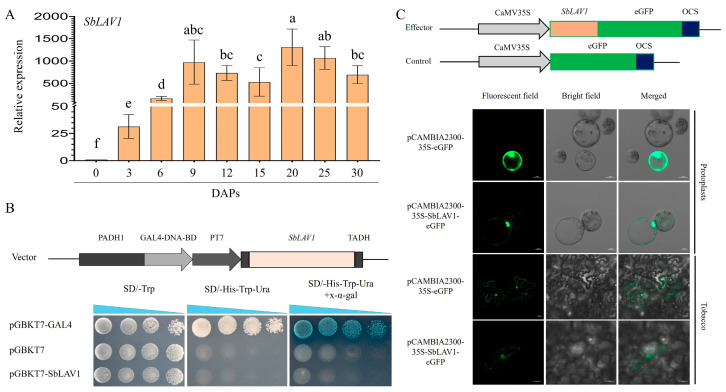
The functional properties analysis of SbLAV1. (**A**) The expression profile of *SbLAV1* among different developmental stages of grains; different letters on the bars refer to the significances at *p* < 0.05, while the same letter refers to non-significance; (**B**) The self-activation analysis of SbLAV1 in yeast strain; (**C**) The sub-cellular location of SbLAV1 in protoplast and tobacco.

Expression pattern analysis of *SbLAV1* in sorghum grain at different developmental stages was separately conducted *via* RT-qPCR for the distinguished high FPKM values. The results showed that *SbLAV1* exhibited high expression levels since 3 DAPs and kept the high expression levels with the relative value > 500 from 9 DAPs to 25 DAPs (Figure 6A), consistent with the observed trends *via* RNA-seq.

The transactivation activity of SbLAV1 was detected *via* the GAL4-based Y2H system. The experiments plasmid pGBKT7-SbLAV1, negative control pGBKT7, and positive control pGBKT7-ZmMYB14 [27] were transformed into yeast strain AH109 and verified by nutrient-deficient culture medium and PCR. The X-α-gal substrate exhibited distinct degradation profiles across sample groups, with blue observed in positive control clones, whereas no color reaction was detected in either negative controls or experimental samples. The results demonstrated that SbLAV1 exhibited no self-activating trans-activity in yeast (Figure 6B). The subcellular localization of SbLAV1 was investigated in both maize leaf protoplast and tobacco. Results of both assays showed that the eGFP signals of SbLAV1 could be found in nuclei and cell membranes, suggesting that SbLAV1 functions in nucleus and cell organelles (Figure 6C). 

### 2.8. SbLAV1-Driven Transcription Activity

**Figure 7:**
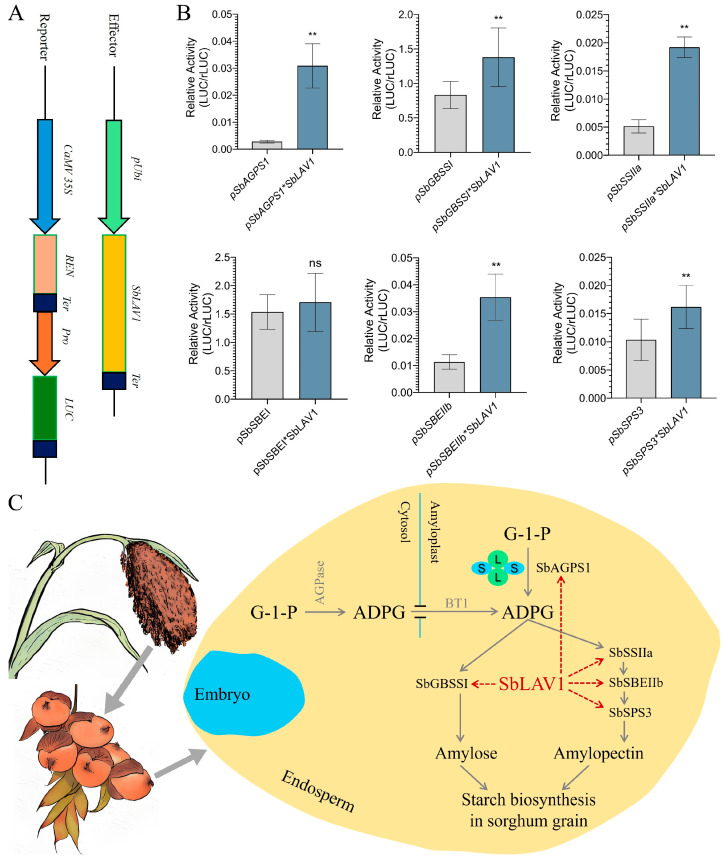
The transcriptional regulation of starch biosynthesis by SbLAV1 in sorghum grain. (**A**) Schematic of experimental vector construction; (**B**) Effect of SbLAV1 on promoter activities of SBRGs in sorghum. ns refers to non-significance, while ** refers to significance at *p* < 0.01; (**C**) the predicted transcriptional regulation mediated by SbLAV1 in sorghum grain, L and S correspondingly refer to the large and small subunits of AGPase, and red dashed arrows present the predicted positive activations of SbLAV1 to sorghum SBRGs.

To further demonstrate that SbLAV1 was involved in the transcriptional regulation of starch synthesis in sorghum grains, we cloned the promoters of six SBRGs, including *SbAGPS1* (1956 bp), *SbGBSSI* (1902 bp), *SbSSIIa* (1917 bp), *SbSBEI* (1869 bp), *SbSBEIIb* (1971 bp), and *SbSPS3* (1758 bp). Co-transformed the *pUbi-SbLAV1* and *pGreenII0800-Pro-Luc* into the maize leaf protoplast, detected the activities of Renilla luciferase (rLUC) and luciferase (LUC), and then calculated the ratio of LUC/rLUC to discover whether SbLAV1 regulated the promoter activity of target SBRGs (Figure 7A). The results showed that SbLAV1 could significantly increase the promoter’s biological activities of *SbAGPS1*, *SbGBSSI*, *SbSSIIa*, *SbSBEIIb*, and *SbSPS3*, while exhibiting a non-significant effect on the promoter of *SbSBEI* (Figure 7B). According to the results of LUC/rLUC, a predicted schema was summarized on the transcriptional regulation of starch biosynthesis by SbLAV1 in sorghum grains (Figure 7C).

## 3. Discussion

### 3.1. The B3 Transcription Factor Family Is Highly Conserved in Plants

The B3 TF family represents a crucial group of TFs in plants, which has been extensively investigated in different plants for its potential functions in plant growth and development, stress responses, and phytohormone signaling pathways [40]. The B3 conserved domain serves as a defining feature for categorizing B3-type TFs, which comprise various subtypes such as B3, Bfil_C_EcoRII_N_B3, and B3_DNA, and each domain exhibits distinct distribution patterns among different B3 proteins [40,50]. Furthermore, B3 TFs may also harbor additional conserved domains, including Auxin_resp, AP2, AUX_IAA, zf-CW, PHD-like, and others [40,51]. 

The B3 family TFs in plants are generally classified into four subfamilies, i.e., ARF (AUXIN RESPONSE FACTOR), LAV (LEAFY COTYLEDON2 [LEC2]–ABSCISIC ACID INSENSITIVE3 [ABI3]–VAL), RAV (RELATED TO ABI3 and VP1), and REM (REPRODUCTIVE MERISTEM) [40]. Genome-wide identification of B3 family members has been systematically conducted across diverse plant species according to advances in genome sequencing, including in *Arabidopsis* [40,52], maize [52], rice [52,53], soybean [54], and castor bean [55]. These comprehensive identification results have provided foundations for elucidating the functional diversity of B3 TFs across the plants.

In the present study, a total of 76 B3 TFs were systematically identified across the sorghum genome. This number is somewhat smaller than those collected by some databases, i.e., 110 in PlantTFDB (https://planttfdb.gao-lab.org/family.php?sp=Sbi&fam=B3, accessed on 18 May 2025), because some B3 genes in the sorghum genome possess more than one transcript. While in the present study, if the target B3 gene possessed two or more transcripts, only the longest version that might cover more complete translations was kept for further analysis. The SbB3 TFs possess subfamilies of ARF, LAV, RAV, and REM (Figure 1A), similar to the B3 TF families of other plants [40,50,54]. Conserved domain analysis revealed distinct conserved domain patterns among sorghum B3 members. Both SbARFs and SbRAVs possessed a single B3-type domain predominantly localized in the C-terminal region of their amino acid sequences (Figure 2B). In contrast, SbREMs contained one or multiple B3-type domains distributed variably along the polypeptide chains (Figure 2B). Furthermore, subfamily-specific auxiliary domains were also identified among the conserved domains, such as SbARFs containing Auxin_resp and AUX/IAA domains, SbLAVs of SbLAV2 and SbLAV3 incorporating zf-CW domains, SbRAV11 containing an AP2 domain, and SbREMs containing Pro-rich domains (Figure 2B). Similar trends are also documented among the B3 family proteins of other plants [40,50,54].

Analyses of conserved motifs and gene structures within sorghum B3 TFs revealed distinct divergence patterns among subfamilies. Specifically, motifs within the same subfamily exhibited conserved spatial distribution and organizational consistency (Figure 2A), aligning with structural domain conservation patterns observed in B3 gene families across other plant species [40,50,54]. Gene structure analysis further confirmed the differences between sorghum subfamilies associated with results in different plants [40,50,54], indicating the conservation of the B3 family in plants. Meanwhile, the differences observed among these B3 subfamilies suggest functional differences among distinct B3 subfamilies.

### 3.2. Differential Expression Patterns of Sorghum B3 Genes

The tissue-specific expression patterns of genes are commonly related to their biological functions. While the gene expression is directly regulated by transcriptional mechanisms, it may be linked to specific signaling pathways. In this study, *cis*-elements were predicted within the 2000 bp sequences upstream of the TSS or ATG of 76 sorghum B3 genes. Four major groups of *cis*-elements were identified, i.e., light-responsive elements, phytohormone-responsive elements, growth/development-related elements, and stress-responsive elements (Figure 3). Previous studies in *Dimocarpus longan* documented widespread *cis*-elements associated with abscisic acid (ABA) response, gibberellin (GA) response, salicylic acid (SA) response, light response, and defense/stress responses within the promoters of B3 family genes [50]. The distribution of these *cis*-elements in sorghum B3 gene promoters suggests that transcriptional regulation of B3 is by distinct signaling pathways. Research results in soybeans and other plants have confirmed that B3 family genes can be induced by plant hormones and cold stress [50,54]. 

Expression profiling of sorghum B3 family genes across diverse tissues revealed marked divergence in expression patterns among different subfamilies (Figure 4). Such differential expression patterns among B3 genes are observed across other plant species [51,54], rather than being exclusive to the sorghum B3 family. Notably, several B3 gene expressions in sorghum were identified as undetectable in normal conditions, suggesting their potential activation under specific induction or developmental stages (Figure 4 and Figure 5). Among the sorghum B3 subfamilies, four genes belonging to *SbLAV*s were highly expressed in developing sorghum grains, while most of the other B3 genes were observed to possess moderate or low expression in the developing grains (Figure 4). Conversely, genes of *SbARF*s and *SbREM*s demonstrated ubiquitous transcriptional activity across all examined tissues, with minimal variations in transcript abundance levels (Table S2, Figure 4). This expression of heterogeneity implies substantial functional diversification within the sorghum B3 family, suggesting the potential regulatory roles of their members.

### 3.3. SbLAV1 Protein Potentially Regulates the Starch Biosynthesis in Sorghum Grains

Starch is a crucial storage product in the grains of cereal crops, closely related to both grain quality and yield performance. Numerous studies documented that transcriptional regulation is an important pattern for starch biosynthesis and accumulation in crops [20]. For instance, TFs of cereals-rice (*Oryza sativa*) [23,56], wheat (*Triticum aestivum*) [33,34,57], and maize (*Zea mays*) [25,29,58,59] have been identified to participate in the transcriptional regulation of grain starch biosynthesis. Previous research on sorghum grains also suggested that TFs, i.e., SbDof21 and NAC-type proteins, may be involved in the transcriptional regulation of starch biosynthesis [38,39]. All these TFs have been found to have relatively abundant transcripts in grains, and some TFs even exhibited co-expression with key genes for starch biosynthesis. In this study, a genome-wide analysis of the B3 TF family in sorghum revealed that genes of *SbLAV1* and *SbLAV5* exhibited grain-specific expression patterns (Figure 4B). Results from co-expression analysis, an effective strategy to identify candidate TFs involved in starch biosynthesis in rice and maize [21,60], further demonstrated that *SbLAV1* co-expresses with multiple SBRGs at different development stages in sorghum grains (Figure 4E), implying important potential regulation functions in developing grains in sorghum.

B3 family members possess diverse functions in plants. For example, in *Arabidopsis*, ABI3 regulates ABI1 to control cell length in the primary root elongation zone [61], REM16 promotes flowering time *via* directly binding to the promoters of *SOC1* and *Flowering Locus T* (*FT*) [62], and VAL1 represses the *FT* to regulate the floral transition [63]. Furthermore, B3 family TFs also play important roles in seed development, dormancy, and the synthesis and storage of nutrients. For example, AtFUS3, AtLEC2, and AtABI3 can directly regulate grain development and play critical roles in lipid metabolism and starch biosynthesis [41,55]. Similarly, ZmAFL4 and ZmABI19, as the LAV subfamily of B3 proteins, directly regulate the transcription of key genes involved in seed development and grain filling in maize [48,49]. Meanwhile, *ZmABI19* was induced by ABA and could also participate in the regulation of nutrient accumulation *via* directly binding *cis*-elements to regulate the transcription of TFs highly expressed in grains [49,64]. In this study, we further confirmed that SbLAV1 localizing to the nucleus could also activate the promoter activities of multiple key SBRGs in sorghum (Figure 6 and Figure 7B) and predicted a transcriptional regulation pathway related to starch biosynthesis mediated by SbLAV1 (Figure 7C). These results provide additional evidence for SbLAV1 participating in the transcriptional regulation of starch biosynthesis in developing sorghum grain.

Additionally, beyond *SbLAV1*, diverse phytohormone-related binding *cis*-elements were also observed within the 2000 bp upstream ATG or TSS of other *SbB3*s (Figure 3), suggesting the potential transcription regulation of the corresponding TFs to SBRGs in sorghum grains. Furthermore, only a relatively narrow region of 5’UTR was covered in the present study, while for the functional dissection of some genes, the regulatory factors might locate in a wide span of 5’UTR [65], such as the enhancer of *etb1.2* for *tb1* in maize, which is located >41 Kb upstream of the TSS of *tb1* [66]. In the further functional dissection of target *SbB3*s, a wider range of 5’UTR regions needs to be covered to avoid missing potential regulatory elements.

## 4. Materials and Methods

### 4.1. Plant Materials and Growth Condition

The sorghum line BTx623, provided by the Rice and Sorghum Research Institute, Sichuan Academy of Agricultural Sciences (Luzhou, China), was grown on the lab farm with normal irrigation and fertilization. The seeds were collected at different developmental stages, including 0 DAP, 3 DAPs, 6 DAPs, 9 DAPs, 12 DAPs, 15 DAPs, 20 DAPs, 25 DAPs, and 30 DAPs. All the collected seed samples were immediately frozen in liquid nitrogen and stored at −80 °C.

### 4.2. Identification of B3 TFs from the Sorghum Genome

To identify putative B3 TFs in the sorghum genome, we performed a conserved domain analysis using the Pfam database (accession: PF02362; http://pfam.xfam.org/, accessed on 15 March 2023). The B3 domain profile was retrieved from Pfam and subsequently employed to screen the *Sorghum bicolor* reference genome assembly (v3.1.1) available through Ensembl Plants (https://plants.ensembl.org/, accessed on 15 March 2023). For the retrieved sorghum B3 genes, for those that possessed more than one transcript, only the versions with the longest transcripts were kept for the corresponding analysis, including related coding products. SMART and NCBI-CDD were used to confirm putative genes [67,68]. The incomplete and redundant sequences were removed by sequence alignment.

### 4.3. Evolutionary and Synteny Analysis of Sorghum B3 TFs

To elucidate the evolutionary relationships among sorghum B3 TFs, multiple sequence alignment was first performed using MUSCLE (v3.8.425). Additionally, all *Arabidopsis* B3 TFs were also downloaded from the TAIR database (https://www.arabidopsis.org/browse/gene_family/B3binding, accessed on 16 March 2023). After removing six TFs whose names contained “-like”, i.e., RAV-like1, the rest were kept for joint evolutionary analysis of B3 TFs from both sorghum and *Arabidopsis*. Neighbor-joining (NJ) trees were generated through MEGA 11.0 with 1000 bootstrap replicates and applying the Poisson model with pairwise deletion of gaps [69]. MCScanX (https://github.com/wyp1125/MCScanX, accessed on 16 March 2023) was used to identify segmental and tandem duplication events. Synteny relationships were visualized using TBtools (v2.031) [70].

### 4.4. Gene Structure Analysis, Motif and Conserved Domain Identification

The exons and introns of B3 genes were analyzed through TBtools (v2.031) based on the genome annotation information [70]. Conserved motifs in sorghum B3 TFs were identified by the online MEME Suite analysis (v5.5.8, https://meme-suite.org/meme/), and the results were visualized by TBtools (v2.031) [70]. The conserved domains of sorghum B3 proteins were predicted through SMART (http://smart.embl-heidelberg.de/, accessed on 18 March 2023).

### 4.5. Analysis of Cis-Elements

Sequences of 2000 bp upstream of the transcription start site (TSS) or ATG of all B3 genes in sorghum were extracted from the reference genome file by TBtools (v2.031). PlantCARE (http://bioinformatics.psb.ugent.be/webtools/plantcare/html, accessed on 21 March 2023) was used to predict the *cis*-elements of each B3 gene, and TBtools (v2.031) was used for result visualization.

### 4.6. Transcriptome Data Analysis

FPKM values were retrieved from the RNA-seq datasets that were previously generated in our lab from multiple tissues to investigate the expression patterns of sorghum B3 genes [38]. The expression levels were normalized based on the FPKM values, and heatmaps were generated through the TBtools (v2.031) [70].

### 4.7. Co-Expression Analysis with Sorghum SBRGs

Based on the FPKM values of RNA-seq data from multiple tissues [38], Pearson correlation coefficients (PCCs) between B3 genes and starch biosynthesis-related genes (SBRGs) were calculated to discover the co-expression trends of B3 genes and SBRGs. The correlation matrix was visualized by Cytoscape (v.3.6.1, http://www.cytoscape.org) to illustrate the co-expression network.

### 4.8. Cloning and RT-qPCR Analysis

Total RNA was extracted from developing grains at 0 DAP and then 3 DAPs, 6 DAPs, 9 DAPs, 12 DAPs, 15 DAPs, 20 DAPs, and 25 DAPs through TRIzol reagent (Invitrogen, Carlsbad, CA, USA) following standardized cryopreservation protocols. First-strand cDNA was synthesized from 1.5 µg total RNA using the PrimeScript^TM^ RT reagent kit with gDNA Eraser (TaKaRa, Dalian, China) according to the manufacturer’s instructions. The first-strand cDNA was used as the template for gene cloning and RT-qPCR analysis.

KOD enzymes (Toyobo, Osaka, Japan) with high fidelity were used to clone *SbLAV1* and nine other selected B3 genes in sorghum. The amplified products of ten genes were constructed into the pMD-19T vector (TaKaRa, Dalian, China) and further verified by sequencing. The primers used for the gene cloning were listed in Table S3.

Two genes, *SbActin1* and *SbEif4α*, were used as the internal controls for RT-qPCR assays. All reactions were performed *via* the Bio-Rad CFX96 real-time system with three biological replications, and the relative expression levels of each replication were calculated through the method of 2^−∆∆CT^ based on the expression of both *SbActin1* and *SbEif4α* [38,39]. The statistical differences among the relative expression levels at 0DAP to 30DAP were detected through multiple comparisons of Duncan test *via* R (v3.2.0). The primers used for the RT-qPCR analysis were listed in Table S3.

### 4.9. Vector Construction

The pBI221 (Clontech, Takara, Dalian, China) was modified for the transient overexpression assay. The *Ubiquitin* (*Ubi*) promoter replaced the original 35S promoter. *SbLAV1* was subcloned into pBI221 that was driven by the *Ubi* promoter, with *BamH*I and *Sac*I restriction sites engineered into the PCR primers for directional cloning. Vector of pCAMBIA2300-35S-eGFP was constructed for sub-cellular localization analysis [38,39]. *SbLAV1* was amplified with the primers containing restriction sites of *BamH*I and *Xba*I without a termination codon. The PCR products of *SbLAV1* were subcloned into pCAMBIA2300-35S-eGFP to form the fusion protein with enhanced green fluorescent protein (eGFP). The two-hybrid yeast system of GAL4 was applied to reveal the self-activating activity of SbLAV1 in yeast. The SbLAV1 was sub-cloned into the pGBKT7 vector by using the sense primer with *Nde*I and the anti-sense primer with *BamH*I. All the primers for the vector construction were listed in Table S3, and the vectors were constructed *via* ClonExpress^R^ MultiS One Step Cloning Kit (Vazyme, Nanjing, China).

### 4.10. Functional Property Analysis of SbLAV1

The subcellular localization of SbLAV1 was examined by using maize leaf protoplasts [29,39]. The pCAMBIA2300-35S-SbLAV1-eGFP recombinant construct was introduced into protoplasts *via* polyethylene glycol (PEG)-mediated transformation with Ca^2^^+^ co-treatment. Transformed protoplasts were maintained in darkness for 16 h to allow gene expression prior to fluorescence analysis. Cellular fluorescence patterns were captured using an LSM 800 fluorescence microscope equipped with Airyscan (Zeiss, Jena, Germany) under blue excitation light at 488 nm. 

The pGBKT7-SbLAV1 recombinant plasmid was transformed into a yeast strain, AH109, to assess transcriptional activation potential. The monoclones were subsequently inoculated into 2 mL of SD/-Trp liquid medium and cultured to logarithmic phase (OD600 = 0.6–0.8) with orbital shaking at 150 rpm. For transcriptional activation verification, all the monoclones were plotted onto quadruple-selective media (SD/-Trp-His-Ura) containing 24 µg/mL X-α-gal under dark conditions of 28 °C for three days.

### 4.11. Dual-Luciferase Assay in Maize Leaf Protoplast

The *pBI221-ubi-SbLAV1* and the *pGreenII0800*-*Pro*-LUC were used to detect the relationship between promoter activities and SbLAV1. The promoters of SBRGs were subcloned into the pGreenII0800-LUC vector to drive the expression of Luciferase (Luc) [38]. The *pBI221-Ubi-SbLAV1*:*pGreenII0800-Pro-Luc* (1:1) was set as the experimental group, and *pGreenII0800-Pro-Luc* as the control group. All the constructs were transformed into the maize protoplast according to Xiao et al. [29]. The activities of LUC and Renilla luciferase (rLUC) were measured *via* the Dual Luciferase Reporter Gene Assay Kit (Yeasen, Shanghai, China) and analyzed *via* GloMax_2020 (Thermo Fisher Scientific, Waltham, MA, USA). The LUC/REN ratio was calculated to reveal the relationship between experimental and control groups. Three independent experiments were performed, and each independent experiment consisted of three replicates. The significances of SbLAV1 to the promoter activities of SBRGs were detected by *t*-test *via* R (v3.2.0).

## 5. Conclusions

A total of 76 sorghum B3 family genes (*SbB3*s) were identified across 10 chromosomes, which can be divided into four sub-families of ARF, RAV, LAV, and REM. Multiple *cis*-elements were detected within the promoter regions of *SbB3*s, and all *SbB3*s exhibited different expression patterns among different sorghum tissues, among which four genes of *SbLAV1*/*5*/*4*/*6* showed grain-specific expression patterns. Case dissection confirmed the co-expression trends of *SbLAV1* with 15 key SBRGs and revealed the promotion of SbLAV1 to the promoter activities of five SBRGs, implying the potential critical roles of *SbB3*s in starch biosynthesis and grain development in sorghum.

## Data Availability

The data presented in this study are available at https://www.ncbi.nlm.nih.gov/bioproject/866867 (accessed on 11 April 2023), accession number PRJNA866867.

## Supplementary Materials

The following supporting information can be downloaded at: https://www.mdpi.com/article/10.3390/plants14111701/s1, Figures S1 and S2. Alignment of the B3 domain sequences of LAV sub-family. Figure S3. Alignment of the B3 domain sequences of RAV sub-family. Figure S4. Alignment of the first B3 domain sequences of REM sub-family. Figure S5. The RT-qPCR analysis of sorghum B3 genes in sorghum grains at different development stages by using SbEif4α as an internal. Different letters on the bars refer to the significances at *p* < 0.05, while the same letter to non-significance. Table S1. Gene information and physicochemical properties of sorghum B3 family. Table S2. The FPKM values of all sorghum B3 genes. Table S3. Primer sequences in the present study.

## Abbreviations

The following abbreviations are used in this manuscript:

AGPase	ADP-glucose pyrophosphorylase
SS	Starch synthase
SBE	Starch-branching enzyme
DBE	Debranching enzymes
SP	Starch phosphorylase
ADPG	ADP-glucose
BT1	Brittle1
LAV	LEC2 [LEAFY COTYLEDON2]-ABI3 [ABSCISIC ACID INSENSITIVE3]–VAL
ARF	AUXIN RESPONSE FACTOR
RAV	RELATED TO ABI3 and VP1
REM	REPRODUCTIVE MERISTEM
DAP	Days after pollination
SBRGs	Starch biosynthesis related genes
