# Supplementary figures and images for: Genome-Wide Dissection of Sorghum B3 Transcription Factor Family Identifies SbLAV1 as a Critical Transcriptional Regulator of Starch Biosynthesis in Developing Sorghum Grains
Source: Plants (Basel). 2025 Jun 3;14(11):1701. doi: 10.3390/plants14111701 (PMC12157820; doi:10.3390/plants14111701)

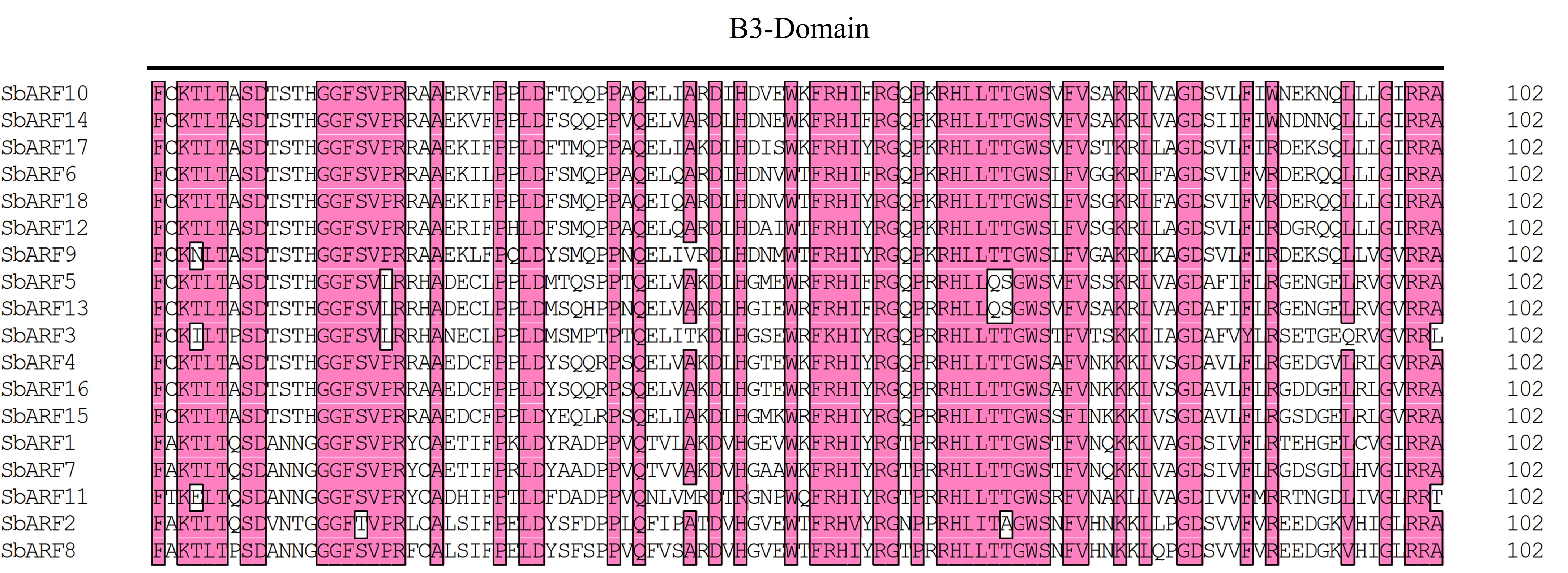

Supplement: Supplementary file 1 [file plants-14-01701-s001.zip › Figure S1.jpg]

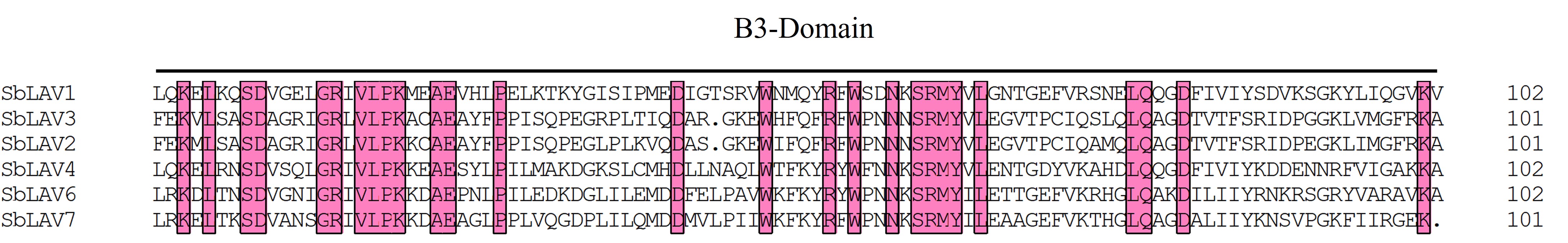

Supplement: Supplementary file 1 [file plants-14-01701-s001.zip › Figure S2.jpg]

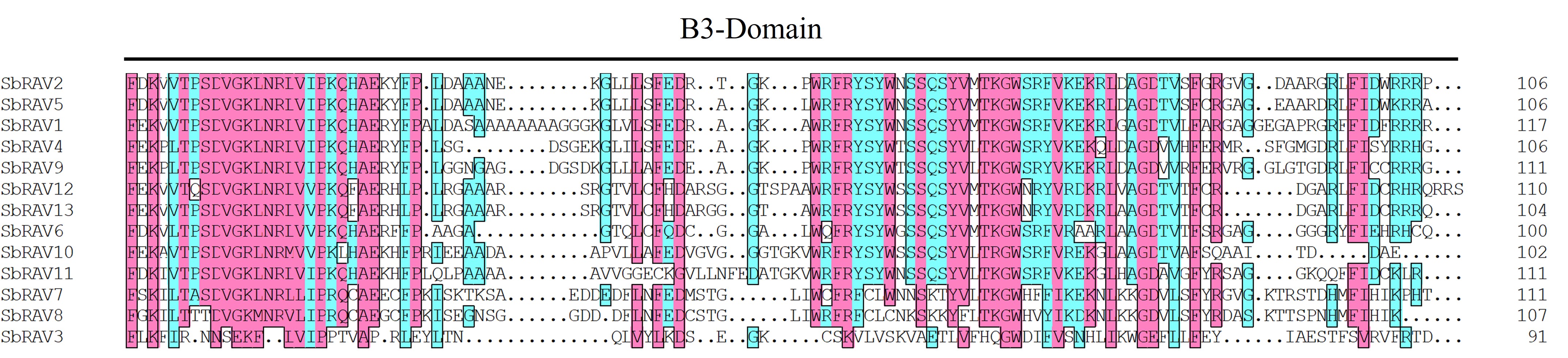

Supplement: Supplementary file 1 [file plants-14-01701-s001.zip › Figure S3.jpg]

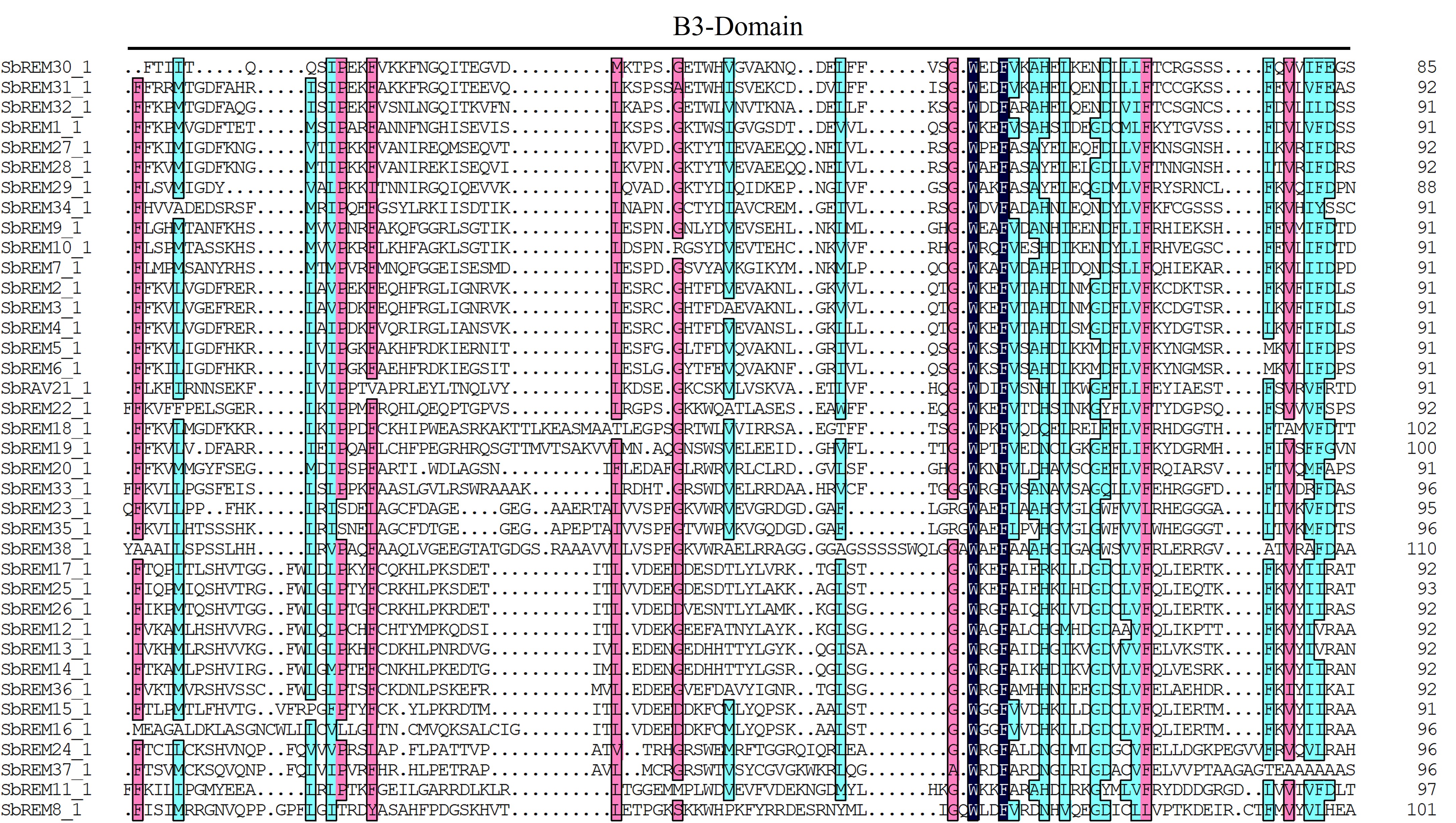

Supplement: Supplementary file 1 [file plants-14-01701-s001.zip › Figure S4.jpg]

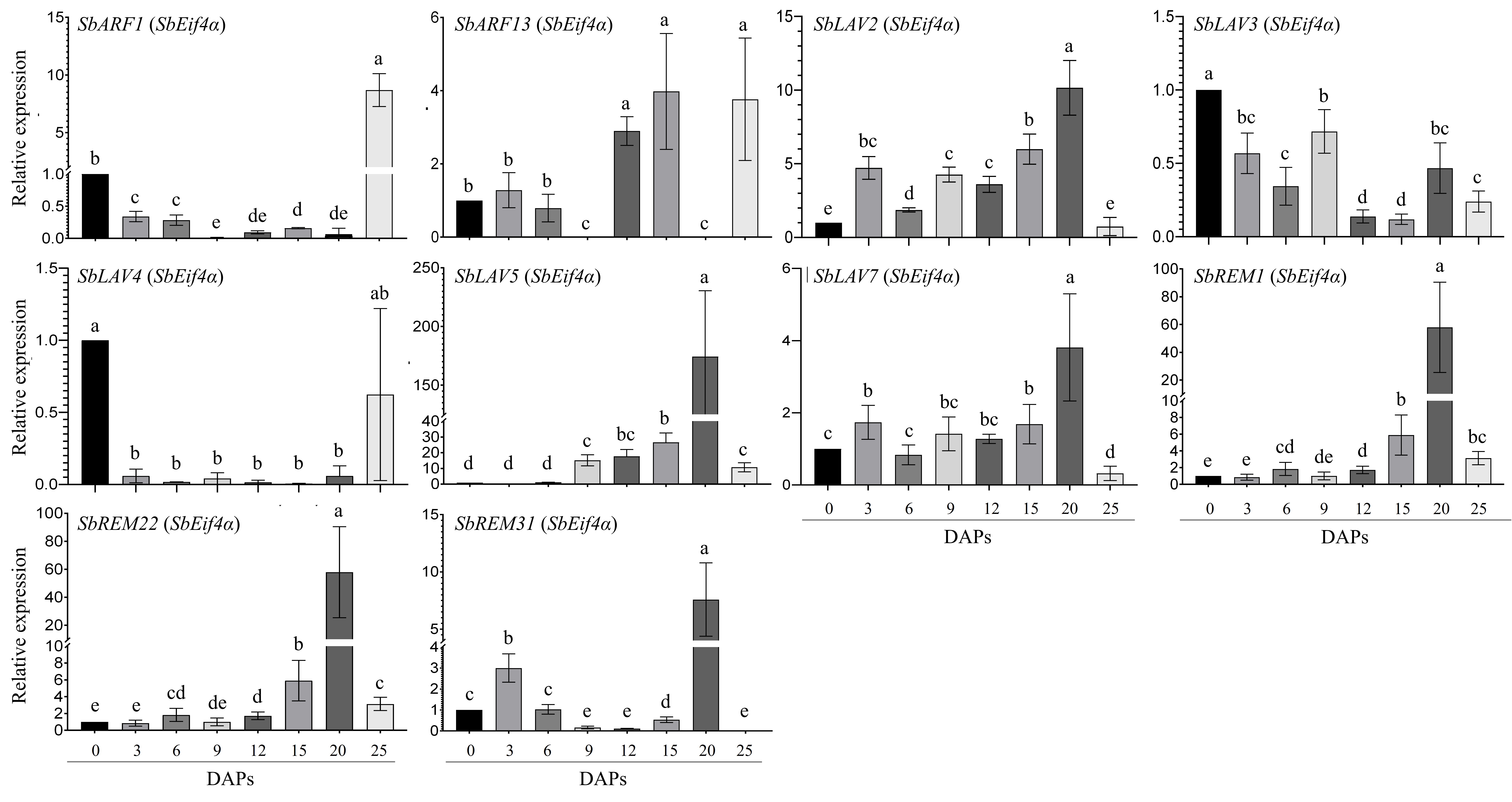

Supplement: Supplementary file 1 [file plants-14-01701-s001.zip › Figure S5.jpg]
